# knnAUC: an open-source R package for detecting nonlinear dependence between one continuous variable and one binary variable

**DOI:** 10.1186/s12859-018-2427-4

**Published:** 2018-11-22

**Authors:** Yi Li, Xiaoyu Liu, Yanyun Ma, Yi Wang, Weichen Zhou, Meng Hao, Zhenghong Yuan, Jie Liu, Momiao Xiong, Yin Yao Shugart, Jiucun Wang, Li Jin

**Affiliations:** 1 Ministry of Education Key Laboratory of Contemporary Anthropology, Department of Anthropology and Human Genetics, School of Life Sciences, Fudan University, Shanghai, China; 2 Six Industrial Research Institute, Fudan University, Shanghai, China; 3 State Key Laboratory of Genetic Engineering, Collaborative Innovation Center for Genetics and Development, School of Life Sciences, Fudan University, Shanghai, China; 4 Department of Computational Medicine & Bioinformatics, University of Michigan, Ann Arbor, MI USA; 5 Shanghai Public Health Clinical Center, Fudan University, Shanghai, China; 6 Key Laboratory of Medical Molecular Virology of MOE/MOH, Shanghai Medical School, Fudan University, Shanghai, China; 7 Department of Digestive Diseases of Huashan Hospital, Collaborative Innovation Center for Genetics and Development, Fudan University, Shanghai, China; 8 Human Genetics Center, School of Public Health, University of Texas Houston Health Sciences Center, Houston, TX USA; 9 Human Phenome Institute, Fudan University, Shanghai, China; 10 Unit on Statistical Genomics, Division of Intramural Division Programs, National Institute of Mental Health, National Institutes of Health, Bethesda, MD USA

**Keywords:** Open source, R package, Nonlinear dependence, One continuous variable, One binary dependent variable, AUC, Association analysis

## Abstract

**Background:**

Testing the dependence of two variables is one of the fundamental tasks in statistics. In this work, we developed an open-source R package (knnAUC) for detecting nonlinear dependence between one continuous variable X and one binary dependent variables Y (0 or 1).

**Results:**

We addressed this problem by using knnAUC (k-nearest neighbors AUC test, the R package is available at https://sourceforge.net/projects/knnauc/). In the knnAUC software framework, we first resampled a dataset to get the training and testing dataset according to the sample ratio (from 0 to 1), and then constructed a k-nearest neighbors algorithm classifier to get the yhat estimator (the probability of y = 1) of testy (the true label of testing dataset). Finally, we calculated the AUC (area under the curve of receiver operating characteristic) estimator and tested whether the AUC estimator is greater than 0.5. To evaluate the advantages of knnAUC compared to seven other popular methods, we performed extensive simulations to explore the relationships between eight different methods and compared the false positive rates and statistical power using both simulated and real datasets (Chronic hepatitis B datasets and kidney cancer RNA-seq datasets).

**Conclusions:**

We concluded that knnAUC is an efficient R package to test non-linear dependence between one continuous variable and one binary dependent variable especially in computational biology area.

**Electronic supplementary material:**

The online version of this article (10.1186/s12859-018-2427-4) contains supplementary material, which is available to authorized users.

## Background

In statistics, dependence is any statistical relationship (causal or not) between two random variables or bivariate data. Correlation is any statistical relationships involving dependence which it is often used to refer to the degree to which the two variables have a linear relationship to each other. Random variables are dependent if they do not satisfy a mathematical property of probabilistic independence [1, 2]. And mutual information can be applied to measure dependence between two variables [3].

The logistic regression or logit regression is a regression model in which the dependent variable is categorical [4]. Logistic regression was developed by statistician David Cox in 1958 [5, 6]. Logical regression estimates the probability by using a logical function, which is the cumulative logistic distribution, to measure the relationship between the categorical variable and one or more independent variables. Other common statistical methods for assessing the dependence between two random variables include distance correlation, Maximal information coefficient (MIC), Kolmogorov-Smirnov (KS) test, Hilbert-Schmidt Independence Criterion (HSIC) and Heller-Heller-Gorfine (HHG). Distance correlation, was proposed by Gabor J Szekely (2005), is a measure of statistical dependence between two random variables or two random vectors. It is zero if and only if the random variables are statistically independent [7, 8]. The maximal information coefficient (MIC) is a measure of the degree of the linear or nonlinear association between two variables, X and Y. The MIC belongs to the maximal information-based nonparametric exploration (MINE) class of statistics [3]. The maximal information coefficient uses binning as a means to apply mutual information on continuous random variables. The Kolmogorov–Smirnov (KS) test quantifies a distance between the empirical distribution function of the sample and the cumulative distribution function of the reference distribution, or between the empirical distribution functions of two samples [2, 9]. HSIC was an independence criterion based on the eigen-spectrum of covariance operators in reproducing kernel Hilbert spaces (RKHSs), consisting of an empirical estimate of the Hilbert-Schmidt Independence Criterion [10]. Heller-Heller-Gorfine (HHG) is a powerful test that is applicable to all dimensions, consistent against all alternatives, and is easy to implement [11].

We had previously proposed an algorithm named continuous variance analysis (CANOVA) [12], which was inspired by the analysis of variance (ANOVA) of continuous response with a categorical factor. In the CANOVA framework, we first proposed a concept of “neighborhood value” based on the value of X, and then we use the permutation test to find the *P* value of the observed “with neighborhood variance” [12].

To further detect the nonlinear dependence between one continuous variable and one binary variable, an open-source R package (knnAUC, https://sourceforge.net/projects/knnauc/) was developed. In the knnAUC framework, the AUC estimator based on a k-nearest neighbors classifier was calculated firstly [13, 14], and then the significance of the AUC based statistic was further evaluated. In order to investigate the feasibility of knnAUC, the false positive rates [15] and statistical power [16] of knnAUC and the other seven commonly used correlation coefficients were evaluated in the simulation studies. To evaluate the performance of knnAUC in real datasets, we further compared their performance in both one real chronic hepatitis B (CHB) dataset [17] and one kidney cancer RNA-seq (transcriptome sequencing) dataset [18, 19].

## Implementation

### Summary

The key idea of knnAUC is based on a comparison test of area under curve (AUC) for Response Operating Characteristic (ROC). Mason and Graham calculated the *p* value based on the Mann-Whitney U statistics [20, 21]. The p value addresses the null hypothesis [20, 21]: variable X cannot be used to discriminate between “Y = 1” and “Y = 0”, that is to say, AUC equals 0.5.

For one continuous variable X and one binary variable Y, we firstly resampled a dataset to get the training and testing dataset according to the sample ratio (sample number of training dataset/sample number of total dataset, range from 0 to 1), and then constructed a k-nearest neighbors algorithm classifier [13, 14] to get the yhat estimator (the probability of y = 1) of testy. At last, we calculated the AUC estimator and tested whether the AUC estimator is greater than 0.5.

### Pseudocode for knnAUC

*Input: one continuous variable X and one binary variable Y, both are of length N.*

*Parameter:*

- *x, a vector containing values of a continuous variable (X).*
- *y, a vector containing values of a binary (0 or 1) discrete variable (Y).*
- *ratio, the training sample size ratio (from 0 to 1),* ratio = (sample number of training dataset)/(sample number of total dataset).
- *kmax, a positive integer, we’ll automatically find the best parameter k for knn between 1 and kmax. The best number of nearest neighbors (k) is determined automatically using leave-one-out cross-validation, subject to an upper limit (kmax).*

**Table:** 

Software Framework:	
1. resample dataset by row without replace **(**resample only once**): **data = data (y, x) if (trainy has both 0 and 1) {train = data (select number_of_rows*ratio)} if (testy has both 0 and 1) {test = data (remaining rows)} 2. calculate yhat by knn:yhat = knn (train, test, kmax) 3. calculate the AUC estimator and test whether AUC is greater than 0.5:result = auc.test(testy, yhat) 4. return AUC estimator and pvalue:auc = result.auc, pvalue = result.pvalue	

## Results

### Results from simulation study

To estimate power of different methods, we simulated nine simple functions of the binary logistic regression model (including binomial distribution function, linear function, quadratic function, sine function and cosine function), as shown in Table 1. The independent variable X follows normal distribution (mean = 0, standard deviation = 1). Nine simple functions were simulated between logit (P(Y = 1|X)) and X, including constant functions (Y follows Bernoulli distribution), linear functions, quadratic functions, sine functions and cosine functions. Five algorithms were chosen as benchmarks: Logistic regression, Distance correlation coefficient, MIC, Kolmogorov–Smirnov test and CANOVA. To calculate the false positive rate, the data was simulated 10,000 times. The statistical power was calculated by repeating 1000 times. The sample size (N) is set as 100. It is worth noting that we fixed the knnAUC parameters (default parameters, ratio = 0.46, kmax = 100) used in simulation study. And MIC also has a bias/variance parameter (the ‘alpha’ parameter in the minerva implementation): the maximal allowed resolution of any grid [3]. Reshef et al. also found that different parameter settings (α = 0.55, c = 5) can make the calculation faster and do not significantly affect performance [22]. For the sake of simplicity, here we only use the default parameters of the MIC (α = 0.6, c = 15).

**Table 1:** Simulation power in nine simple simulation functions

*N* = 100, X~N (0,SD^2), SD = 1	Logit	Distance	MIC	KS	Canova	knnAUC
Y~ Bernoulli distribution (*p* = 0.5)	0.050	0.047	0.027	0.048	0.043	0.048
logit (P(Y = 1|X)) = X + 1	**0.989**	0.979	0.627	0.947	0.406	0.648
logit (P(Y = 1|X)) = (0.25^*^X + 1)^2 + 1	**0.302**	0.277	0.034	0.236	0.062	0.118
logit (P(Y = 1|X)) = sin (pi^*^X + 1) + 1	0.042	0.107	0.266	0.186	0.199	**0.306**
logit (P(Y = 1|X)) = sin (2^*^pi^*^X + 1) + 1	0.050	0.055	0.183	0.073	**0.196**	0.192
logit (P(Y = 1|X)) = sin (3^*^pi^*^X + 1) + 1	0.045	0.050	0.137	0.053	**0.170**	0.120
logit (P(Y = 1|X)) = cos (pi^*^X + 1) + 1	0.037	0.108	0.265	0.197	0.186	**0.291**
logit (P(Y = 1|X)) = cos (2^*^pi^*^X + 1) + 1	0.050	0.052	0.179	0.078	0.175	**0.179**
logit (P(Y = 1|X)) = cos (3^*^pi^*^X + 1) + 1	0.046	0.048	0.123	0.056	**0.168**	0.111

The bold means the first place result of all methods compared. * means multiplication operator

To test the Type-I error rate of benchmarked methods, the data was simulated 10,000 times to estimate the false positive rate (Table 1, Y~ Bernoulli distribution). The Type-I error of all methods are less than 0.05, indicating their nominal levels are well controlled (Table 1). In the comparison with other non-constant functions in the simulation data, we showed some interesting findings in Table 1: (1) in the case of linear correlation, the logistic regression was the most powerful method, knnAUC also performed well. (2) in the case of non-linear correlation, the performance of knnAUC and CANOVA were two of the most powerful method, especially in the function of a high degree of shock/non-linear situation. (3) knnAUC was superior to the MIC algorithm in most cases.

In order to detect the performance of knnAUC and other algorithms, different variance levels in the simulation were performed (mean = 0, standard deviation = 1/3, 1/2, 2 and 3), and the power across different levels of variance was reported (shown in Additional file 1). From Additional file 1, we arrived to the following conclusions after adding different variance to Y: (1) When the variance level was low (standard deviation = 1/3, 1/2), most of the methods performed poorly. However, knnAUC and Distance were two of the most powerful method among all non-linear functions, logistic regression had a higher power in linear functions. (2) When the variance level was high (standard deviation = 2, 3), most of the methods in the complex sine/cosine functions was less powerful, but knnAUC and CANOVA had higher power than other methods. For simple linear dependence, most of the methods were relatively efficient. Therefore, to obtain a higher statistical effect, when the relationship between the two random variables is linear or relatively simple, we recommend the logit regression. When the relationship is non-linear or complex, knnAUC and CANOVA are better choices for exploring the dependence structure of the binary class of dependent variables and the continuity independent variables.

### Results from chronic hepatitis B (CHB) dataset

We compared the knnAUC algorithm with the other seven algorithms using a real gene expression dataset for chronic hepatitis B (CHB) dataset, which included 122 samples and gene expressions with three clinical parameters [17]. The level of dependence among inflammation grades, gene expressions and clinical parameters (ALT, AST and HBV-DNA) were tested in large-scale CHB samples [17].

We have one binary dependent variable Y for the degree of inflammation of the liver (G). Age, gender, ALT, AST, and HBV were all standardized values. These five variables were clinical physiologic indexes. The expression levels of 17 significant genes [17] were our X variables. The significance level is preset to be 0.05. It is worth noting that we used the knnAUC default parameters (ratio = 0.46, K = 100) in the CHB dataset. For simplicity, the other algorithms were also applied the default parameters (especially for MIC, α = 0.6, c = 15).

The *p*-value comparison of all methods for chronic hepatitis B (CHB) dataset [17] is shown in Table 2. All knnAUC results were realized in the R environment (https://sourceforge.net/projects/knnauc/), CANOVA was realized in the C++ environment, the other four benchmarks were calculated using the R packages ‘energy’ [23], ‘Hmisc’ [24] and ‘minerva’ [25]. All results were calculated on a desktop PC, equipped with an Intel Core i7–4790 CPU and 32 GB memory.

**Table 2:** Corresponding p-values of liver inflammation grades in CHB dataset (α = 0.05)

Variables	knnAUC	Logit	Distance	MIC	KS	CANOVA
Gender	7.889E-01	7.527E-01	7.094E-01	8.841E-04	1.00E + 00	5.132E-01
Age	6.957E-01	4.304E-01	4.633E-01	1.696E-01	3.54E-01	6.023E-01
AST	**1.387E-05**	**4.524E-03**	**2.000E-05**	**4.581E-01**	**5.90E-06**	**3.729E-03**
ALT	**2.180E-04**	**8.211E-05**	**1.000E-05**	**4.229E-01**	**3.36E-06**	**1.574E-04**
***HBV***	**6.121E-03**	6.775E-01	1.827E-01	2.557E-01	1.19E-01	9.440E-02
DLX3	7.755E-01	**2.928E-02**	**4.196E-02**	1.877E-01	6.61E-02	7.607E-01
ALPK1	**2.007E-02**	**1.458E-03**	**2.220E-03**	2.719E-01	**9.67E-03**	2.619E-01
YBX1	**2.791E-02**	**7.759E-05**	**1.100E-04**	**3.419E-01**	**3.95E-03**	3.390E-01
ZNF75A	2.584E-01	1.288E-01	**3.924E-02**	2.662E-01	**4.24E-02**	2.619E-01
SPP2	**6.084E-04**	8.177E-02	**3.031E-02**	2.681E-01	**3.09E-02**	9.435E-02
TTLL4	3.332E-01	5.029E-01	5.182E-01	2.411E-01	6.73E-01	2.620E-01
TTLL7	1.350E-01	2.789E-01	3.477E-01	2.097E-01	3.43E-01	6.025E-01
***AGAP3***	**3.300E-02**	7.963E-01	8.173E-02	2.611E-01	1.74E-01	1.386E-01
DCTN4	**4.869E-03**	**4.212E-02**	**1.367E-02**	2.534E-01	**8.61E-03**	2.619E-01
IGF1R	7.545E-01	7.296E-01	9.058E-01	1.714E-01	8.44E-01	6.850E-01
PRDX2	6.649E-01	1.120E-01	1.898E-01	2.281E-01	4.14E-01	6.024E-01
NKAPL	9.824E-01	8.817E-01	6.992E-01	2.598E-01	7.37E-01	**3.871E-02**
NRXN1	7.167E-01	9.583E-01	9.895E-01	1.670E-01	9.82E-01	9.165E-01
NXF2	1.473E-01	9.902E-01	8.698E-01	1.899E-01	7.14E-01	9.166E-01
Pou2f2	5.958E-01	3.176E-01	3.898E-01	2.034E-01	3.79E-01	7.607E-01
SIRPB2	9.394E-01	3.853E-01	6.399E-01	1.771E-01	9.04E-01	8.766E-01
TRD	3.733E-01	6.533E-01	1.965E-01	2.445E-01	1.10E-01	8.766E-01

If MIC> 0.31677, then *p* value < 0.050004564

Variable Y: G on behalf of liver inflammation grades, two categories

Variable X: age; gender; ALT, AST, HBV_DNA is the value after standardization; 17 primitive gene expression

The significant values are shown in bold; the significant variables detected only by knnAUC are shown in bold italics

Then, a literature review for validation of each significant gene was performed using pubmed (https://www.ncbi.nlm.nih.gov/pubmed/). In the dependence study of inflammation grades of hepatitis (Y), two significant variables were only detected by knnAUC algorithm, shown in Table 2, one is clinical variable HBV-DNA and the other is AGAP3 gene. HBV-DNA is an important standard to assess pathological features (such as the inflammation level G) and determine prognosis for hepatitis B virus (HBV)-infected patients. The prognosis and outcome of treatment for chronic hepatitis B virus (HBV) infection are predicted by levels of HBV DNA in serum [26]. What’s more, AGAP3 was reported having predictive power for inflammation grades of chronic hepatitis B [17]. ALT, DLX3, ALPK1, YBX1 and DCTN4 were detected by a variety of algorithms at the same time. NKAPL was specifically detected by the CANOVA algorithm. Serum parameters (e.g. alanine amino transaminase [ALT] and aspartate amino transaminase [AST]) are utilized to access the damage of liver and HBV viral infection [27]. In our previous principal component analysis (PCA) research, DLX3, ALPK1, YBX1, DCTN4 and NKAPL have a strong ability to predict inflammation grades [17].

### Results from the kidney cancer study

To further evaluate the performance of the knnAUC algorithm, we also compared knnAUC with the other seven algorithms using a real RNA-seq dataset of kidney cancer, which included 604 samples (532 cancer cases, 72 normal controls) and 20,531 genes. We tested the correlation level between X (20,531 gene expression data) and Y (whether it was kidney cancer) [18, 19]. At the same time, the computing time of each algorithm was compared. The significance level was preset to be 2.435342e-06 (Bonferroni correction). It is worth noting that we used the knnAUC default parameters (ratio = 0.46, K = 100) in kidney cancer dataset. For simplicity, other algorithms also applied the default parameters (especially MIC, α = 0.6, c = 15), which were shown in Table 3.

**Table 3:** Comparison of all methods in kidney cancer dataset (the significance level α = 2.435e-06)

Kidney cancer dataset	knnAUC	Logit	MIC	KS	Distance	CANOVA
Unique genes reported in Pubmed	4	2	1	**6**	2	1
The number of unique genes	65	293	14	**566**	124	18
Significant gene number	8453	9633	8081	**11,915**	10,946	5901
Computing time (seconds)	0.0912	0.0068	0.0052	**0.0033**	258.9717	19

The bold means the first place results of all methods compared. The Computing time was recorded between 1 gene and 604 samples

In the real kidney cancer data, the comparison of the power and computing time of different methods are shown in Table 3. In Additional file 2, we only listed the genes detected by knnAUC which were not detected by other methods. At the same time, genes that can only be detected by other methods were listed in Additional file 3.

From Table 3, it can be seen that the Spearman correlation coefficient can detect the most number of significant genes (11,629 genes, α = 0.05 / 20,531) in real kidney cancer RNA-seq data. But the KS test detected the most number of unique genes. And interesting observation made is that the computing time of knnAUC was significantly faster than distance and CANOVA. To further compare the features of each method and to explore the biology relevance of the detected genes, “significant” genes that were uniquely detected by each method (other methods failed to detect positive) were chosen as the “target gene set”. And then a literature review was performed for the sake of validating each gene in the pubmed database.

**Fig. 1:**
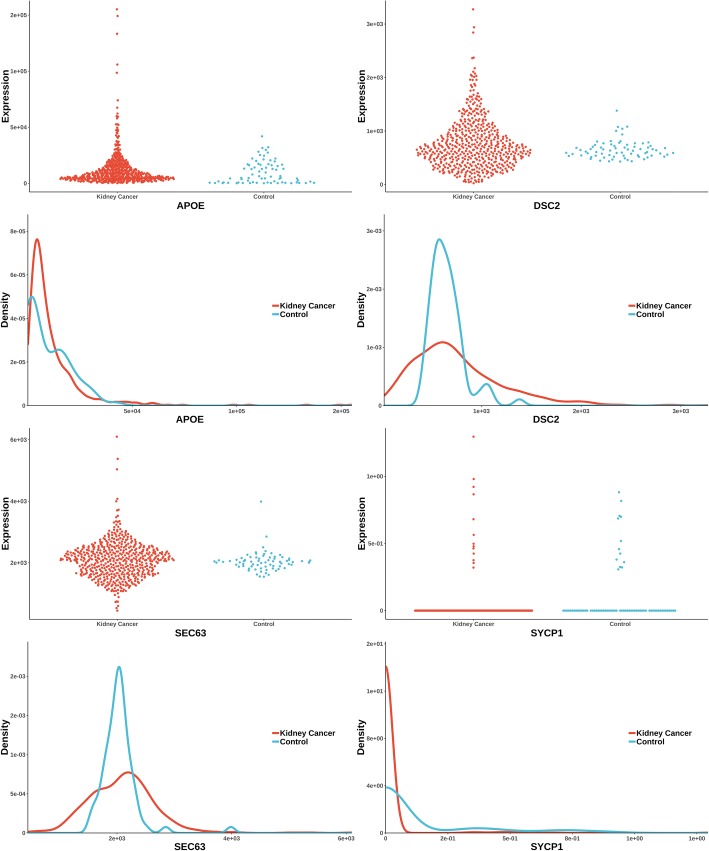
Gene expression (reported significant genes detected only by knnAUC) between kidney-cancer and normal groups

**Fig. 2:**
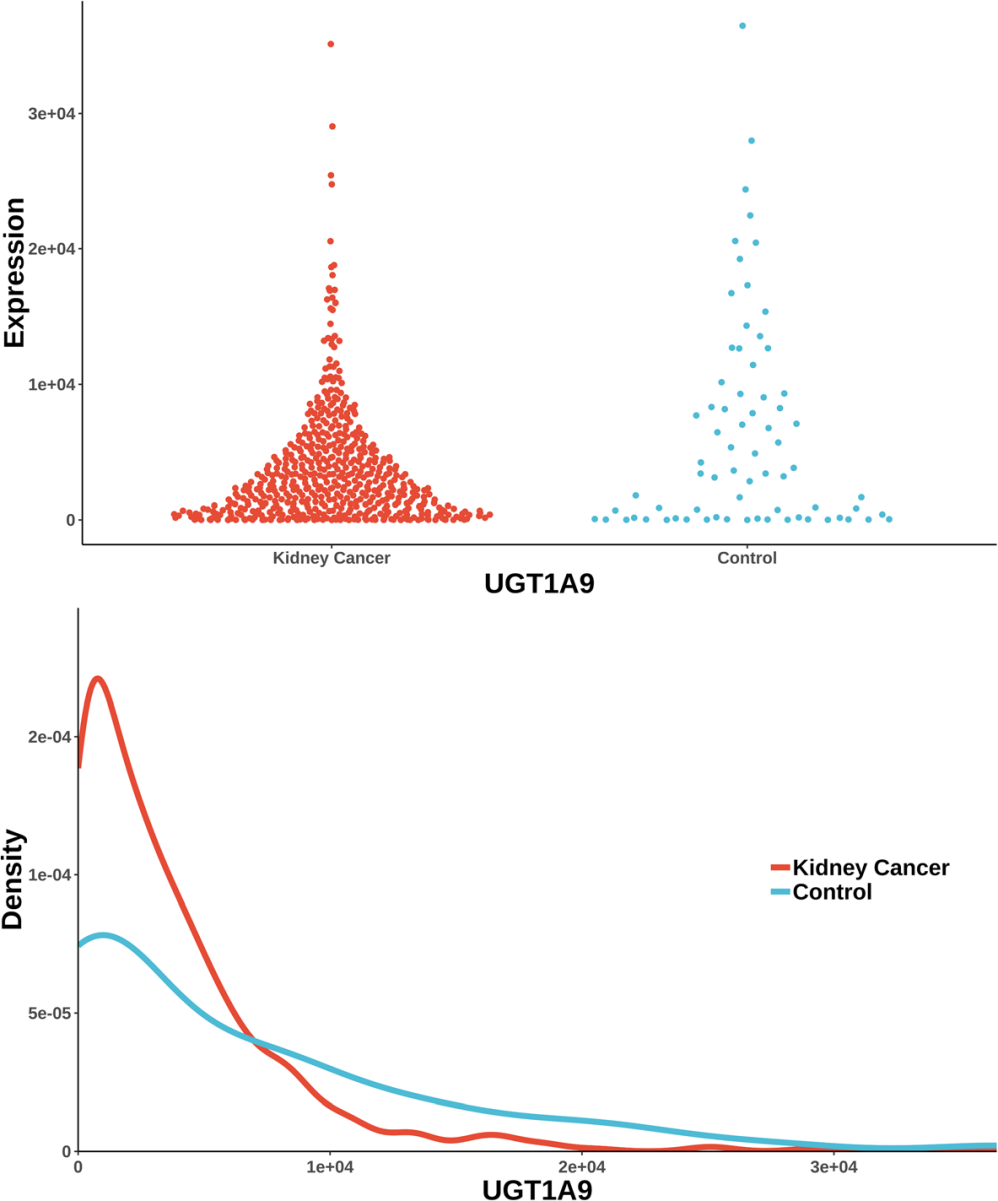
Gene expression (reported significant genes detected only by CANOVA) between kidney-cancer and normal groups

**Fig. 3:**
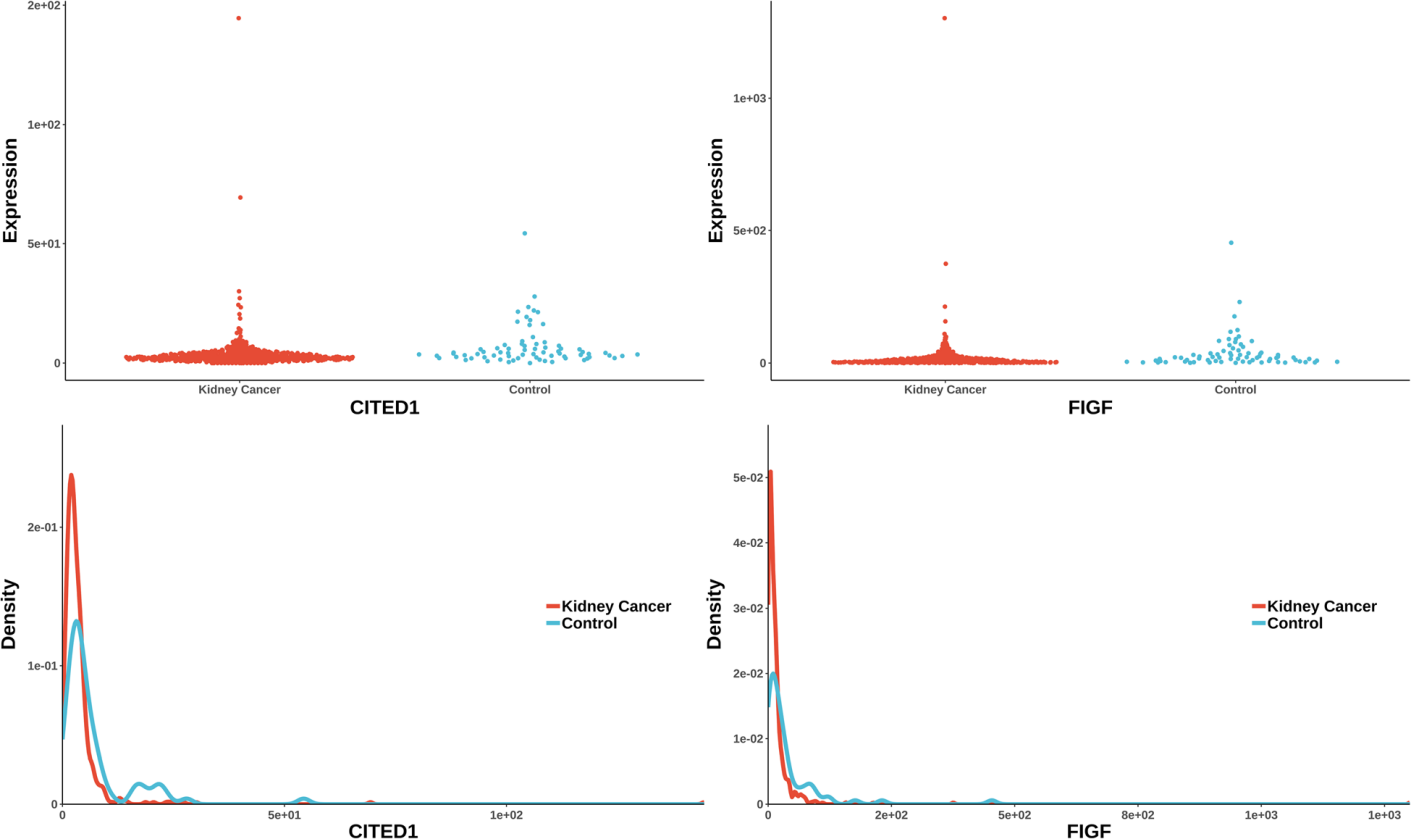
Gene expression (reported significant genes detected only by distance) between kidney-cancer and normal groups

**Fig. 4:**
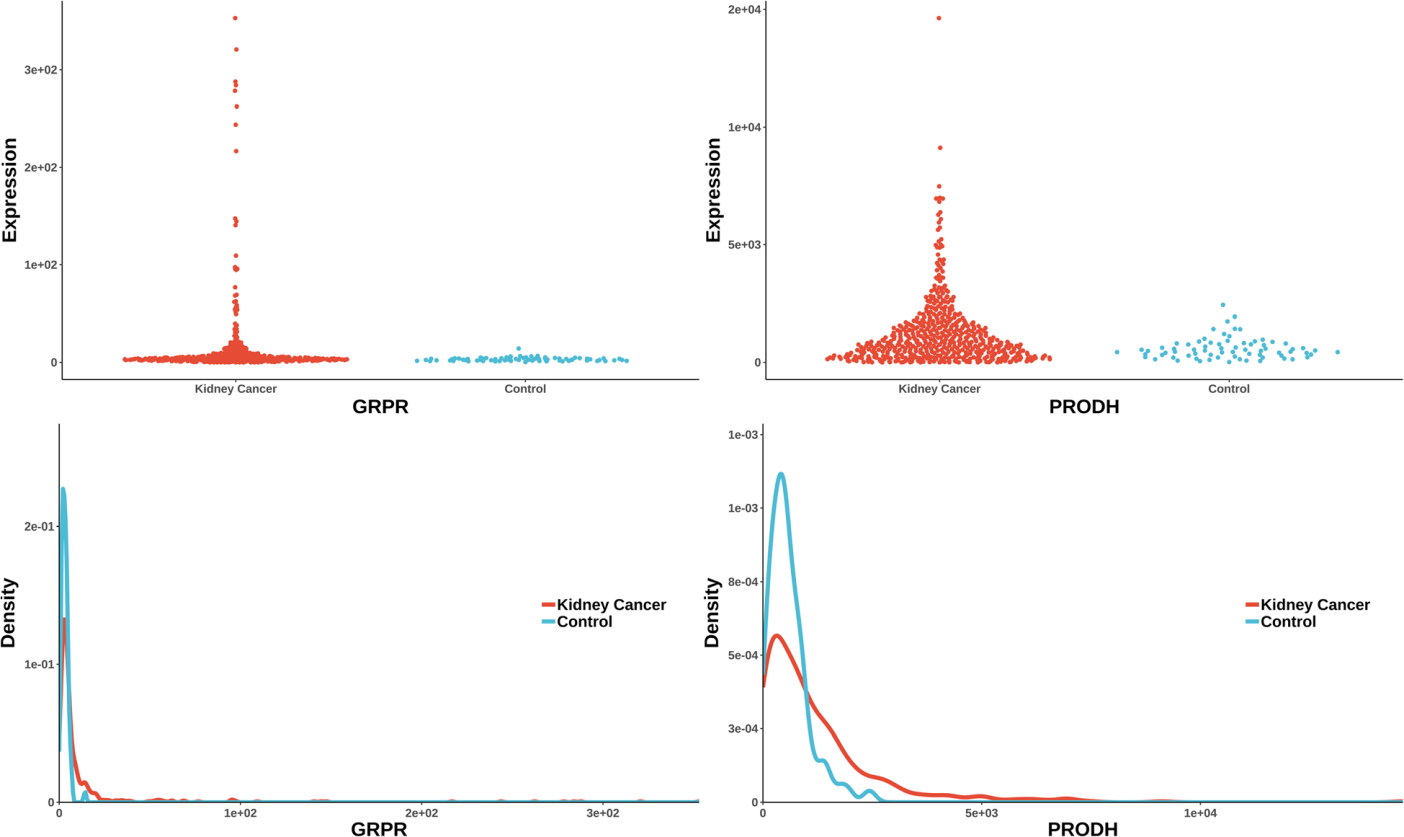
Gene expression (reported significant genes detected only by logistic regression) between kidney-cancer and normal groups

**Fig. 5:**
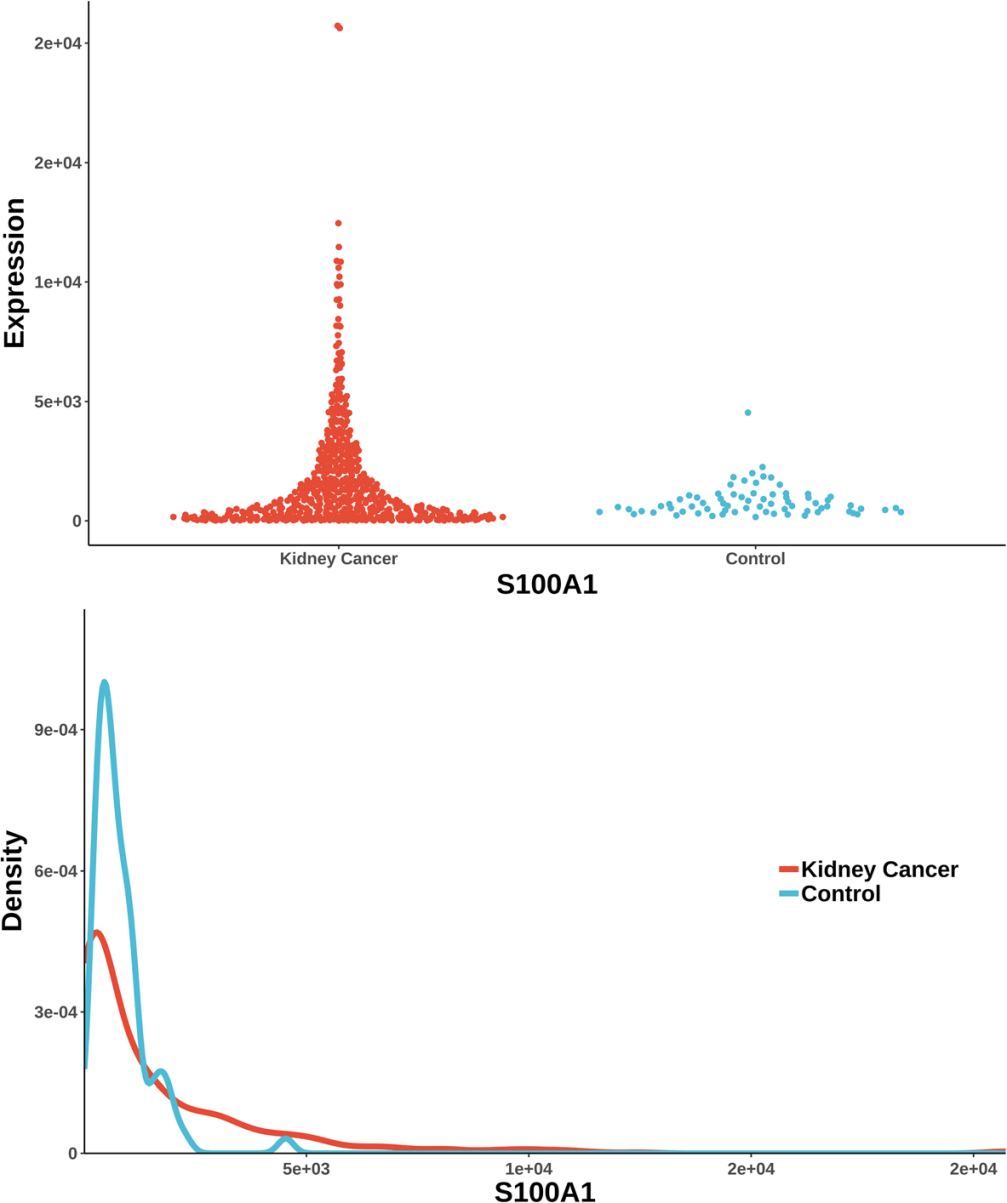
Gene expression (reported significant genes detected only by MIC) between kidney-cancer and normal groups

**Fig. 6:**
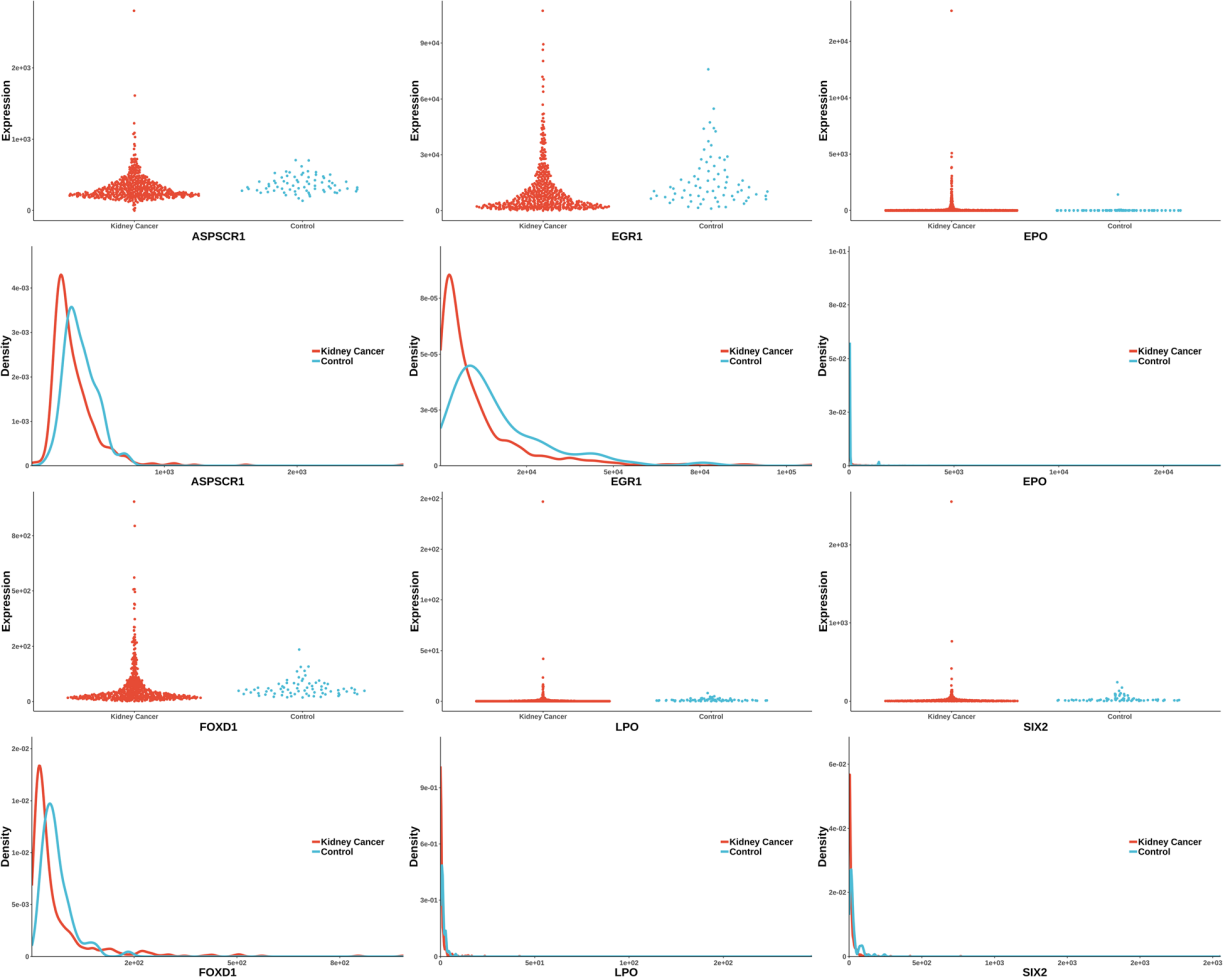
Gene expression (reported significant genes detected only by KS) between kidney-cancer and normal groups

The uniquely significant genes detected by knnAUC and the corresponding *P*-values of all methods are shown in Additional file 2. And genes reported in pubmed (indicating that there is an abstract in Pubmed concerning a relationship with kidney cancer and the gene) are shown in Additional file 2 and Fig. 1 (Scatterplot and probability density distribution). Similarly, the uniquely significant genes found by other methods are shown in Additional file 3 and the genes reported in pubmed are showed in Fig. 2, 3, 4, 5 and 6.

From the unique set of genes detected by knnAUC (Additional file 2), four genes, APOE, DSC2, SEC63 and SYCP1 were reported to be relevant to renal cancer (Fig. 1). A functional region of APOE could increase renal cell carcinoma susceptibility in a two stage case-control study [28]. DSC2 is associated with development and progression of renal cell carcinoma (RCC) [29]. SEC63 is associated with polycystic kidney disease [30, 31]. And copy-number gain of SYCP1 in human clear cell renal cell carcinoma predicts poor survival [32]. Although the distributions of these genes have almost the same mean value and different curvature of the density distribution function, the AUC values of these genes’ prediction models are significantly higher than 0.5, which could be detected by knnAUC method.

UGT1A9 (identified in Additional file 3**,** Fig. 2) were the unique gene (also reported in pubmed database) detected by CANOVA. A significant decrease glucuronidation capacity of neoplastic kidneys versus normal kidneys was related with reduced UGT1A9 and UGT2B7 mRNA and protein expression [33].

Two unique genes (also reported in pubmed database) were detected by distance correlation. They were CITED1 and FIGF (identified in Additional file 3**,** Fig. 3). CITED1 confers stemness to Wilms tumor and enhances tumorigenic responses [34]. FIGF was related with the development of kidney in murine [35]. The two unique genes detected by logistic regression were GRPR and PRODH (identified in Additional file 3**,** Fig. 4). As a receptor for gastrin-releasing peptide (GRP), GRPR promotes renal cell carcinoma by activating ERK1/2 pathway together with GRP [36]. PRODH is among a few genes induced rapidly and robustly by P53, the tumor suppressor [37, 38]. MIC detected one gene, S100A1 (identified in Additional file 3**,** Fig. 5). HNF1β and S100A1 are useful biomarker for distinguishing renal oncocytoma and chromophobe renal cell carcinoma [39].

Six unique genes (also reported in pubmed database) were detected by KS test. They were SIX2, EPO, ASPSCR1, FOXD1, EGR1 and LPO. SIX2 is activated in renal neoplasms and influences cellular proliferation and migration [40]. EPO is related to the development of renal cell carcinoma [41]. A total of five TFE3 gene fusions (PRCC-TFE3, ASPSCR1-TFE3, SFPQ-TFE3, NONO-TFE3 and CLTC-TFE3) have been identified in RCC tumors and characterized at the mRNA transcript level [42]. FOXD1 is an upstream regulator of the renin-angiotensin system during metanephric kidney development [43]. MAML1 acts cooperatively with EGR1 to activate EGR1-regulated promoters, which could also have implications for the development of renal cell carcinoma [44]. Compared to normal renal cortex, the LPO induction period was markedly increased in renal-cell carcinoma [45, 46].

## Discussion and conclusions

Recently, correlations among inflammation grades, gene expressions and clinical parameters (serum alanine amino transaminase, aspartate amino transaminase and HBV-DNA) were analyzed based on a large-scale CHB (chronic hepatitis B) samples [17]. The gene expressions with three clinical parameters in 122 CHB samples was analyzed by improved regression model and principal component analysis [17]. We found that significant genes, such as DLX3, ALPK1, YBX1, DCTN4, NKAPL, ZNF75A, SPP2 and AGAP3 (shown in Table 2), related to clinical parameters have a significant correlation with inflammation grades.

Among all the benchmarked methods, knnAUC detected four unique genes related to renal cancer in pubmed database. Two of these genes were reported to be associated with renal cell carcinoma (RCC). MACC1 and DSC2 are related to the prognosis of RCC [29, 47]. The up-regulation of PDE2A methylation level was reported to promote the development of renal kidney papillary cell carcinoma (KIRP) [48]. Finally, NMD3 has been associated with the suppression of Wilms’ tumor through gene-specific interaction with GRC5 [49].

The non-linear dependence in our study is on the raw scale between one continuous variable and one binary variable, and other transformations will also be considered in our future studies. Theoretically, any machine learning algorithm could be the kernel function of the AUC based independence test we’ve developed. We also tested the performance of random forest [50], support vector machines [51] and generalized boosted models [52] as the kernels, however, they are not as powerful as knnAUC. And k-NN is a classic non-parametric method in machine learning area. But k-NN fails in case of the curse of dimensionality [53]. The curse of dimensionality in the k-NN basically means that Euclidean distance is not helpful in the presence of high dimensions because all vectors are almost equidistant to the search query vector. To avoid overfitting, we only resampled the dataset once which is equivalent to “an independent randomized trial” in statistics. Another advantage of knnAUC is that, it is robust with its two parameters, ratio (the training sample size ratio) and kmax (automatically find the best parameter for knn between 1 and kmax). The knn algorithm was realized by RWeka package [14]. The ratio and kmax don’t significant influence the knnAUC performance. However, they may influence the computing time. For computational efficiency, using default parameters (ratio = 0.46 and k = 100), knnAUC could have competitive results. knnAUC is rather stable when the sample size is large enough (like > 100, we used knnAUC to recalculate Table 1 for 100 times in Additional file 4). And we may sometimes change the parameter ratio when the sample is extreme unbalanced (Additional file 5). For example, when you have too much cases such as 80~ 90% of total samples, you may want to set ratio = 0.1 or 0.2 to get more training samples in knnAUC method. When the average proportion of cases (Y = 1) was above 0.87, we found that the best parameter ratio was almost always 0.1 in Additional file 5. On the other hand, when the sample is not so extreme unbalanced (60~ 70% samples are cases), knnAUC performed well with the default parameters (ratio = 0.46) in Additional file 5. In practice, we can use grid search to tune the two parameters to improve power. For example, the parameter ratio can be tuned from 0.1 to 0.9 by 0.1, and the parameter kmax can be tuned from 2 to sample size by 1 to maximize detection power.

Several methods were proposed to identification of genes related to a certain kind of cancer [54, 55]. In this article, the gene expression datasets are used to explain the purpose of our knnAUC method: detecting non-linear dependence biological signals between one continuous variable X and one binary variable Y. Furthermore, we could quantize the forecast skills of X by AUC and test whether it is significantly above 0.5. That is to say, knnAUC could be used to detect non-linear biological signals, which may be validated by further mechanism experiments.

To sum, we developed an open-source R Package to detect dependence between one continuous variable and one binary variable especially under complex non-linear situations. We concluded that knnAUC (https://sourceforge.net/projects/knnauc/) is an efficient R package to test non-linear dependence between one continuous variable and one binary dependent variable especially in computational biology area.

## Availability and requirements

**Project name:** knnAUC.

**Project home page:** https://sourceforge.net/projects/knnauc/

**Operating system(s):** Windows or Linux.

**Programming language:** R.

**License:** GPL-2.

**Any restrictions to use by non-academics:** licence needed.

## Additional files

Additional file 1: The power comparison of simulation study across different variance levels. (XLSX 14 kb)

Additional file 2: The significant (associated with kidney cancer) genes only detected by knnAUC. (XLSX 16 kb)

Additional file 3: The significant (associated with kidney cancer) genes only detected by other methods. (XLSX 106 kb)

Additional file 4: The recalculated (100 times) simulation power of knnAUC with default parameters in nine simple functions. (XLSX 37 kb)

Additional file 5: The simulation power of knnAUC with different ratios in nine simple functions. (XLSX 22 kb)

## Abbreviations

AUC: Area under curve
CHB: Chronic hepatitis
GRP: Gastrin-releasing peptide
HBV: Chronic hepatitis B virus
KIRP: Renal kidney papillary cell carcinoma
knnAUC: K-nearest neighbors AUC test)
MCM3: Minichromosome maintenance 3
MIC: Maximal information coefficient
MINE: Maximal information-based nonparametric exploration
PCA: Principal component analysis
RCC: Renal cell carcinoma
RKHSs: Reproducing kernel Hilbert spaces
ROC: Response operating characteristic
